# Pyroptosis-Mediated Periodontal Disease

**DOI:** 10.3390/ijms23010372

**Published:** 2021-12-29

**Authors:** Mariane Beatriz Sordi, Ricardo de Souza Magini, Layla Panahipour, Reinhard Gruber

**Affiliations:** 1 Department of Oral Biology, Medical University of Vienna, 1090 Vienna, Austria; marianesordi@hotmail.com (M.B.S.); layla.panahipour@meduniwien.ac.at (L.P.); 2 Department of Dentistry, Federal University of Santa Catarina, Florianopolis 88040-900, Brazil; ricardo.magini@ufsc.br; 3 Department of Periodontology, School of Dental Medicine, University of Bern, 3012 Bern, Switzerland; 4 Austrian Cluster for Tissue Regeneration, Donaueschingenstraße, 1200 Vienna, Austria

**Keywords:** pyroptosis, immune response, inflammation, periodontal disease, periodontitis

## Abstract

Pyroptosis is a caspase-dependent process relevant to the understanding of beneficial host responses and medical conditions for which inflammation is central to the pathophysiology of the disease. Pyroptosis has been recently suggested as one of the pathways of exacerbated inflammation of periodontal tissues. Hence, this focused review aims to discuss pyroptosis as a pathological mechanism in the cause of periodontitis. The included articles presented similarities regarding methods, type of cells applied, and cell stimulation, as the outcomes also point to the same direction considering the cellular events. The collected data indicate that virulence factors present in the diseased periodontal tissues initiate the inflammasome route of tissue destruction with caspase activation, cleavage of gasdermin D, and secretion of interleukins IL-1β and IL-18. Consequently, removing periopathogens’ virulence factors that trigger pyroptosis is a potential strategy to combat periodontal disease and regain tissue homeostasis.

## 1. Introduction

In clinical practice, clinicians frequently face situations where the periodontal or peri-implant tissues overreact to a stimulus promoted by dental materials or even do not respond to therapies, leading to inflammation. In these situations, the claim is that the body is not accepting the treatment, rehabilitation, or therapy [1,2,3,4]. Indeed, it is very likely that processes are occurring inside the cells to cause such exacerbated inflammation. However, the root cause of the inflammatory processes and the activated cell pathways that culminate in tissue damage are beginning to be understood. There may be some novel inflammation pathways leading to exacerbated tissue damage that requires the attention of researchers and clinicians.

**Figure 1:**
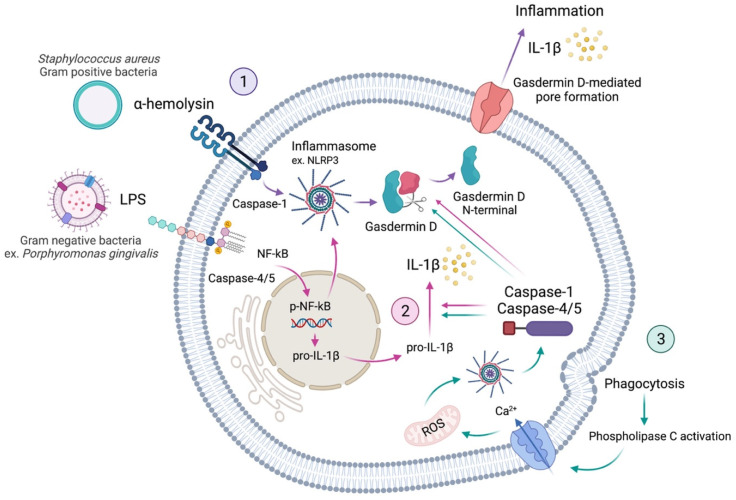
Schematic representation of the pyroptosis activation in a cell. Virulence factors, such as α-hemolysin and LPS, activate inflammasome immune responses by activating caspases. Activated caspase-1 (canonical) and caspases-4/5 (non-canonical) recognise and cleave gasdermin D. The N-terminal fragment, resulting from Gasdermin D cleavage, is responsible for pore formation on the cell membrane. These pores allow the release of interleukins and thus induce the inflammation process called pyroptosis (1, purple arrows). Additionally, activated inflammasomes are responsible for cleaving caspases into subunits, which induce the maturation of pro-inflammatory cytokines, such as pro-interleukin-1β (pro-IL-1β). Activated IL-1β can induce the expression of various genes, including RANKL (receptor activator of p-NF-κB ligand) and activate pyroptosis (2, pink arrows). Furthermore, phagocytosis can lead to pyroptosis through the activation of phospholipase C, which allows the intake of calcium (Ca^2+^), provoking the production of mitochondrial reactive oxygen species (ROS), which can also activate the inflammasome route (3, green arrows) [5,11].

Pyroptosis is a process of cellular self-destruction mediated by caspases. Thus, when pathological or damaging factors stimulate cells, they promote the formation of inflammasomes. Pyroptosis is chiefly mediated by the activation of caspase-1 by the NLRP3 (NOD-, LRR-, and pyrin domain-containing protein 3) inflammasome [5,6]. Caspase-1 cleaves gasdermin D (GSDMD), resulting in cell membrane perforation through the release of the GSDMD N-terminal fragment [5]. This is known as the canonical inflammasome activation of pyroptosis. The non-canonical activation of pyroptosis occurs via the activation of caspases-4 and -5 in humans, or caspase-11 in mice [7,8,9], which also cleave the GSDMD. Thus, the mechanisms of pyroptosis involve different major signalling pathways, all activating the downstream of GSDMD. Finally, cytoplasmic molecules, such as interleukins-1β (IL-1β) and -18 (IL-18), are released from the pores formed by GSDMD and trigger a robust inflammatory response (Figure 1) [2,9,10]. Thus, the occurrence of pyroptosis can be determined by a combination of markers, including the activation of caspases-1, -4, -5, and -11, the cleavage of GSDMD, and the maturation and release of IL-1β and IL-18 [9].

With the discovery of NLRP3, GSDMD, and caspase-1 as significant drivers of pyroptosis, small molecule inhibitors that block these factors functions are expected to emerge for possible treatment of inflammatory conditions [12]. Therefore, this focused review aims to discuss pyroptosis as a catabolic mechanism present, in the oral environment, in the face of exposure to damaging factors, as well as propose it as a target for periodontal therapies. This review further highlights the importance of oral hygiene to avoid accumulating virulence factors that drive pyroptosis in the periodontium.

## 2. Methods

A bibliographical search was performed on MEDLINE/PubMed (via National Library of Medicine) using the following search terms: (innate immune system or immune system or inflammation or inflammatory response or inflammatory process) and (cytokine or interleukin) and (cell death or proptosis) and (virulence factor or virulence factors or hemolysin or nigericin or LPS or lipopolysaccharide) and (inflammasomes or caspase or gasdermin) and (periodontal disease or periodontitis or (periodontal or periodontally or periodontics or periodontic or periodontitis) and (therapeutics or therapies or therapy)). Additionally, a manual search was performed considering the references within the selected articles. The inclusion criteria involved English language articles published up to July 2021, reporting systematic reviews, literature reviews, and in vitro, in vivo, preclinical, and clinical studies on the cell events occurring under the stimuli of different virulence factors that lead to pyroptosis and inflammation. The following exclusion criteria were considered: case reports, protocols, short communications, personal opinions, letters, posters, conference abstracts; full text not available; duplicate data (e.g., dissertations/thesis in which correspondent published articles were available).

Regarding the title and abstract, the evaluation of the potentially relevant articles was accomplished. Selected articles were individually read and analysed, considering the aim of this review. The retrieved variables considered for this review were: authors’ names; journal; publication year; study design; methods; statistical analyses. A software (Zotero, George Mason University, Fairfax County, VA, USA) was used to manage references. A two-phase selection process was performed. In the first phase, a title and abstract reading were conducted to identify potentially eligible studies. In the second phase, full-text reading of eligible articles was carried out. The following data regarding included studies were recorded: study characteristics (author, year, journal of publication), type of study, methods, and main findings/outcomes.

## 3. Results

The initial search strategy retrieved 23 potential studies published from December 2005 to June 2021. The year 2017 was the one that resulted in the most publications (seven studies), followed by 2021 (four studies), and 2016 and 2020 (three studies each year). The manual search resulted in additional information for the present review. One study was a literature review [8], while the 22 remaining articles were in vitro and/or in vivo studies (Table 1 and Table 2).

In the first phase, the studies that analysed other pathways for cell death, such as apoptosis, were excluded from the full reading and inclusion in this review (Table 3). In the second phase, no articles were excluded from the full-text reading. Generally, articles followed similar study designs, methods, purposes, and outcomes. Methods included cell or animal stimulation for pyroptosis, followed by analyses of cell viability, cell morphology, histology, expression of pyroptosis-related proteins, interleukins, and genes. The studies that did not mention pyroptosis but analysed caspases-1, -4, -5, or -11, which are related to pyroptosis, were included in the review [6,7].

## 4. Discussion

The results of this review are divided. Part 1 considers the fundamental concepts and related factors of pyroptosis. Part 2 discusses the results found in Table 1 and Table 2, highlights the clinical relevance of pyroptosis on periodontal diseases, and considers pyroptosis in periodontal therapy.

### 4.1. Part 1—Updated Knowledge of Pyroptosis Inflammatory Pathways

#### 4.1.1. Innate Immune System and Inflammation

Innate immune responses are tightly regulated by various pathways to control infections and maintain homeostasis [29]. Hence, the innate immune system is strongly related to inflammation, which is a host tissue response to an assault commonly triggered by microorganisms or other stimuli such as chemicals, radiation, or trauma, and their released products (i.e., metabolites, endotoxins). Consequently, inflammation is generally related to pathologies, while it is important to highlight that inflammatory reactions provide rapid and early protection against potential aggressor agents. Clinically, host tissues experience several inoffensive inflammatory reactions routinely, as a result of various stimuli, and these responses are not considered infections or diseases. These cases represent situations where the inflammatory response is physiologic and provide early protection from potentially dangerous events [30]. Nevertheless, when the innate immune system is acting against pathogens or other potential dangers, the inflammatory responses are initiated through pattern recognition receptors, phagocytes, dendritic cells, epithelial cells that recognise pathogen-associated molecular patterns (PAMPs), and damage-associated molecular patterns (DAMPs) [31,32,33]. These stimulate the transcription level of interferons, pro-inflammatory cytokines, interleukins, and other essential factors, further leading to the recruitment of other immune cells (such as lymphocytes) to bridge innate and adaptive immune systems [33].

The inflammasome is one of the pathways of the innate immune system, which activates a family of cysteine proteases called inflammatory caspases. These caspases control the immune response by cleaving specific cellular substrates [9,29,34]. Inflammasomes often require activation by danger signals. The inflammasome activation can lead to the release of pro-inflammatory cytokines and, more interestingly, to an inflammatory programmed cell death known as pyroptosis [31,33].

#### 4.1.2. Pyroptosis

Cell death can be categorised by describing the initiating events, the intermediate changes, the terminal cellular events, and its effect on tissue. Each cell death pathway may be distinguished based on these four categories [35]. Apoptosis was the first well-recognised type of cell death, and this “programmed cell death” is broadly applied to several endogenous genetically defined pathways in which the cell plays an active part in its own destruction. However, other cell death paths include autophagy [36], oncosis [37], necroptosis [38,39], NET (neutrophil extracellular traps)osis [40], ferroptosis [41], cytostasis [23], pyroptosis [42,43], among others yet to be discovered. Most of these modalities have specific initiation events In vitro, but not all have well-defined roles in vivo [35].

**Figure 2:**
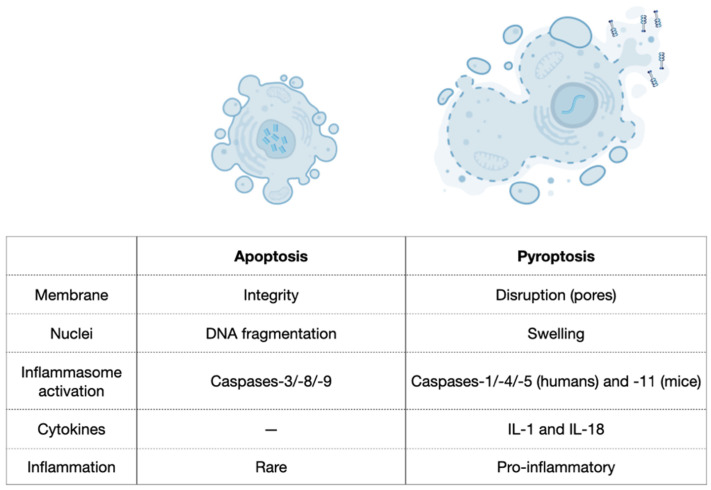
Comparison between apoptosis and pyroptosis. Based on Liu et al., 2018 [47].

Pyroptosis is as an efficient mechanism of bacterial clearance developed by the innate immune system [34]. It was first described in 1992 in macrophages infected with *Shigella flexneri* [44] and later when a similar phenotype was observed after infection with *Salmonella typhimurium* [34,45,46]. Pyroptosis is a process of cellular self-destruction mediated by caspases and, therefore, it was not initially distinguished from the classic apoptosis. However, the mechanisms, characteristics, and outcomes of pyroptosis are very distinct from apoptosis, where the most significant difference is the inflammatory responses (Figure 2) [9,16,34,35,42,47]. Additionally, pyroptosis occurs rapidly, and it is accompanied by the release of numerous pro-inflammatory factors [5]. Thus, the term pyroptosis (from the Greek ‘pyro’, relating to fire or fever, and ‘ptosis’, denoting a falling) is used to describe the remarkable pro-inflammatory process of cell death on pyroptosis [46]. As a clinical example, pyroptosis of peripheral blood mononuclear cells was associated with the severity and the mortality of patients with sepsis [48].

Caspase-1 was first recognised as a protease that processes the inactive precursors of interleukins-1β (IL-1β) and -18 (IL-18) into mature inflammatory cytokines [7,49]. However, caspase-1 activation can result not only in the production of activated inflammatory cytokines but also in rapid cell death characterised by plasma-membrane rupture and release of pro-inflammatory intracellular contents [42,43]. Additionally, DNA cleavage during pyroptosis results from the activity of an unidentified activated caspase-1 nuclease that does not produce the oligonucleosomal DNA fragmentation pattern that is characteristic of apoptosis. DNA cleavage is accompanied by marked nuclear condensation while nuclear integrity is maintained [42,43,50]. Pyroptosis also presents a series of morphological and physiological changes related to the inflammatory response [9,34,51]. Morphologically, small pores (10–15 nm) emerge on the membrane of pyroptotic cells, which change the membrane’s permeability [39]. This event contributes to intracellular bacterial clearance and destroys any niche formed by intracellular bacterial replication because it causes intracellular bacterial exposure to the extracellular compartment, making bacteria more susceptible to antibodies and attacks by phagocytes such as neutrophils [8,9,34]. Additionally, numerous pro-inflammatory cytokines in the cytoplasm are released from the pores to the extracellular matrix [52], promoting cell lysis and death. Finally, the cells burst and an inflammatory response around the dead cells is triggered because of the released cytokines [53]. In contrast, apoptosis involves the controlled dismantling of intracellular components while avoiding inflammation and damage to surrounding cells [47].

When the cell is stimulated, PAMPs and DAMPs promote the formation of inflammasomes. Pyroptosis is mediated by the activation of caspase-1 by the nucleotide-binding domain (NBD) and leucine-rich repeat (LRR)-containing protein 3 (NLRP3) inflammasome [5,6]. Caspase-1 thus cleaves the members of the gasdermin family, including gasdermin D (GSDMD), which subsequently results in the perforation of the cell membrane due to the release of its N-terminal domain [5]. This caspase-1 triggering through NLRP3 is the classical canonical inflammasome activation of pyroptosis, while the non-canonical activation of pyroptosis happens through the triggering of caspases-4 and -5 in humans, or caspase-11 in mice [7,8,9]. Both pathways lead to the cleavage of GSDMD. Therefore, the mechanisms of pyroptosis basically have to involve the downstream of GSDMD, finally leading to pores on the cell membrane, while cytoplasmic molecules, such as IL-1β and IL-18, are released from the pores and provoke a robust inflammatory response (Figure 1) [2,9,10]. Hence, the manifestation of pyroptosis can be determined by a combination of markers, including the activation of caspases-1, -4, -5, and -11, the cleavage of GSDMD, and the activation and release of interleukins IL-1β and IL-18 [9]. Finally, how the pyroptosis pathway is activated is explained by the virulence factors.

#### 4.1.3. Virulence Factors

Healthy cells do not release interleukins when the cells are dying. However, certain virulence factors may activate the inflammasome pathway, leading to cell death and inflammation of the surrounding tissues [54,55]. Different types of classical virulence factors may act on the activation of distinct caspases that will determine the type of cell death.

Staphylococcal α-hemolysin is a bacterial pore-forming toxin produced by *Staphylococcus aureus*, which activates inflammasome activity and caspase-1, thus inducing pyroptosis [56]. *S. aureus* exploits the pro-inflammatory bias of human keratinocytes to activate pyroptosis, which is required for staphylococci to penetrate across the cell membrane [57]. The α-hemolysin role in the pathogenesis of skin infection is well documented [58,59,60]. Still, it remains unclear exactly how these non-motile bacteria invade through the barrier posed by the multiple layers of proliferating and cornified keratinocytes that comprise normal human skin [57].

Nigericin is a microbial toxin produced by *Streptomyces hygroscopicus* that decreases the intracellular potassium (K^+^), which causes caspase-1 activation, leading to pyroptosis [61]. Nigericin binds to K^+^, which is subsequently transported across the plasma membrane as nigericin-K and released on the outside of the cell [62]. Nigericin has been shown to activate NLRP3 inflammasome and induce the release of IL-1β [63,64].

Lipopolysaccharides (LPS) are toll-like receptor (TLR) agonists that are found in the outer membrane of Gram-negative bacteria [30]. LPS have a pro-inflammatory function via modulation of caspases that can cleave GSDMD, the pro-pyroptotic factor (Figure 1) [1,65,66]. Most of the oral pathogens are Gram-negative bacteria, such as *Porphyromonas gingivalis*, *Aggregatibacter actinomycetemcomitans*, *Treponema denticola*, *Fusobacterium nucleatum*, *Tannerella forsythia*; therefore, these pathogens are all able to produce the virulence factor LPS [2,3,5,8,14,15,18,67,68].

#### 4.1.4. Inflammasomes

Inflammasomes are cytosolic multi-protein complexes that perform inflammatory responses when stimulated by pathogens or endogenous hazards [33,69,70]. There are two main classes of inflammasome sensor proteins: (1) nucleotide-binding domain (NBD) and leucine-rich repeat (LRR)-containing proteins (Noll Like Receptors or NLR) and (2) absent in melanoma 2 (AIM2)-like receptors [32]. The oligomerisation of NLR and AIM2-like receptor sensors facilitates the oligomerisation of adapters such as apoptosis-associated, speck-like protein containing a caspase recruitment domain (ASC) [71]. These adaptors trigger the recruitment of effectors, such as pro-caspase-1, that are activated and cleaved into their mature forms [32,33]. Thus, inflammasomes are of central importance to inflammatory processes, as they promote the cleavage of pro-inflammatory cytokines, notably IL-1β and IL-18, through the maturation of caspase-1 [70,72]. Dysregulations or gene mutations of inflammasomes are associated with several auto-inflammatory diseases and cancer [33,73].

Regarding pyroptosis, the NLRP3 inflammasome seems to be the one to act on the activation of caspase-1. Thus, caspase-1 is the essential mediator of inflammasome function and its activity is a direct marker of NLRP3 activation [6,72]. Emerging evidence suggests that the NLRP3 inflammasome can react to a wide range of bacterial ligands, including LPS, bacterial RNA, and peptidoglycans (PAMPs or DAMPs), and plays a pivotal role in the pathogenesis of several diseases, such as rheumatoid arthritis, bone loss, osteomyelitis, periodontal disease, and others, by regulating the inflammatory response. Overexpression of NLRP3 exacerbates inflammatory osteolysis and inhibits calcium deposition in metabolic bone diseases [5,33,69,74]. In this sense, caspases have a close relationship with inflammasomes, once their activity triggers the caspase activation.

#### 4.1.5. Caspases

Caspase is an abbreviation for Cysteine-dependent ASPartate-specific proteASE or cysteinyl aspartate specific proteinase [9]. Caspase-1 is the leading enzyme to mediate the highly inflammatory process known as pyroptosis, which is characterised by rapid cell lysis and the release of pro-inflammatory cytokines [73]. The downstream processes, resulting from caspase-1 activation, are dictated by the cell type and the nature and magnitude of the stimulus received [34,42]. Thus, caspase-1 activation is a host defence mechanism. Pathogens require mechanisms to prevent the potent inflammatory outcome of pyroptosis to persist and cause disease. Likewise, the host should possess means to neutralise pathogen-mediated regulation of caspase-1 activity and successfully control the infection [42]. Nevertheless, although pyroptosis has this protective host response to infectious diseases, exaggerated caspase-1 activation can be detrimental to the surrounding tissues [42].

Caspase-1 is pivotal for pyroptosis. It was originally termed “interleukin converting enzyme” for its well-established role in the cleavage of IL-1β and IL-18 [29]. Upon sensing PAMPs and DAMPs, innate immune cells form inflammasomes that recruit and activate caspase-1, known as the canonical inflammasome pathway. Other inflammatory caspases, such as caspase-4 and -5, directly bind bacterial LPS, triggering pyroptosis, which is the non-canonical inflammasome pathway. However, the non-canonical pathway ultimately leads to canonical inflammasome engagement through caspase-1 activation (Figure 1) [29]. By including specific caspase-1 inhibitors––Ac-YVAD-CHO, for instance, it is possible to discriminate caspase-1 activity from the activity of other caspases, and pyroptosis from other types of cell death [72]. In addition, the activated caspase-1 has a critical role in the cleavage of the GSDMD, another central element of pyroptosis (Figure 1).

#### 4.1.6. Gasdermin D

The GSDM family includes GSDM A, B, C, D, and E, as well as DFNB 59 [75], of which GSDMD is the most important mediator of pyroptosis. GSDMD is cleaved by caspase-1 into two fragments: (1) the N-terminal fragment, and its inhibitory counterpart, (2) the C-terminal fragment. The N-terminal domain can form small pores of 10–15 nm on the cell membrane, that allow the secretion of the cytoplasmatic content, including invading pathogens and pro-inflammatory cytokines. Such cytokines recruit more inflammatory cells to trigger the inflammatory cascade. Additionally, GSDMD pores generate potassium efflux that allow caspase-1 activation through NLRP3 inflammasome (Figure 1) [1,9,29,47]. Thus, GSDMD is a central effector of pyroptosis that has different roles inside the cells, while the most remarkable activity is the formation of pores in the cell membrane, which allow the release of interleukins to the extracellular matrix, then provoking an intense inflammatory reaction.

#### 4.1.7. Interleukins

Pyroptosis is predicted to be pro-inflammatory due to the release of inflammatory cytokines [34]. The cytokines related to pyroptosis are the interleukins IL-1β and IL-18. IL-1β is a potent endogenous pyrogen that stimulates vasodilation, fever, leukocyte tissue migration, immune cell extravasation, and expression of several cytokines and chemokines [34,76]. Macrophages are a prime source of pro-IL-1β that generally depend on caspase-1 for maturation and secretion of the biologically active IL-1β [77]. IL-18 promotes interferon-γ production and activates T cells and macrophages [34,78]. Both IL-1β and IL-18 play crucial parts in the pathogenesis of a range of inflammatory and autoimmune diseases [18,76,78].

The ligation of pattern recognition receptors by PAMPs leads to intracellular production of pro-IL-1β and pro-IL-18. Simultaneous ligation of receptors for DAMPs leads to assembly of NLRP3 and cleavage of pro-caspase-1 into activated caspase-1, which will finally cleave pro-IL-1β and -18 into their mature forms [2,79]. Infections, by many types of intracellular bacteria, stimulate the synthesis of pro-IL-1β, but not its secretion. It has been shown that a second signal, often due to a danger signal (DAMPs) such as extracellular ATP or nigericin, is then able to activate NLRP3 and caspase-1 [77,79]. Hence, pyroptosis is a way to release the processed IL-1β and IL-18 from the cell. Nevertheless, depending on the cell type and stimulus, inflammasome engagement, and caspase-1 activation, IL-1β release may occur in the absence of cell death. Although no cytokines are required for cell death, their production contributes to the inflammatory response generated by cells under pyroptosis [9,42]. The clinical consequences are exacerbated inflammatory reaction, tissue damage, and disease.

#### 4.1.8. Clinical Relevance

Inflammasome mutations can lead to inappropriate caspase-1 activation, which is associated with autoinflammatory syndromes [80]. Moreover, caspase-1 is involved in the pathogenesis of several diseases, including periodontal disease [4], Alzheimer’s disease [81,82], cardiovascular disease [83], rheumatoid arthritis [84], endometriosis [70], and Crohn’s disease [85], all of which are characterised by cell death and inflammation. Caspase-1 deficiency or inhibition protects against cell death, inflammation, and tissue dysfunction, associated with these diseases. Thus, caspase-1 is a potential therapeutic target through specific pharmacological inhibitors [29,42]. However, it is essential to emphasise that caspase-1 is also part of the immune system and thus crucial for protection against virulence factors. Research to identify and characterise novel caspase substrates can also expand the understanding of inflammatory caspases in health and disease. Consequently, research should address how endogenous mechanisms and inhibitors control inflammatory caspase activity. Pyroptosis and other caspase-1-dependent processes are therefore relevant to the understanding of beneficial host responses and medical conditions for which inflammation is central to the pathophysiology of the disease [42].

Considering that pyroptosis is strongly associated with inflammatory diseases and that the virulence factors existing in the oral environment can provoke the exacerbation of the pyroptosis towards strong inflammation and tissue damage, it is likely that pyroptosis is associated with periodontal disease. In the meantime, such inflammasome pathway on the periodontal tissue is poorly explored. Hence, Part 2 of this discussion will argue the role of pyroptosis on periodontal disease.

### 4.2. Part 2—Pyroptosis on the Periodontal Diseases and Periodontal Therapy

The knowledge of pyroptosis in the pathogenesis of periodontitis is evolving. This can be noticed from the articles retrieved from the search strategy applied herein. It is also clear that other areas of medical knowledge besides dentistry focus on understanding pyroptosis-mediated inflammation processes [12,75,85,86,87,88,89,90]. Regarding periodontitis and periodontal therapies, the included articles present similarities regarding methods, type of cells involved, concentration, and kind of pyroptosis stimulation, as the outcomes point to the same direction considering the cellular events. Therefore, the gathered data led to a discussion on the related processes and the clinical relevance of studying pyroptosis in periodontitis.

**Figure 3:**
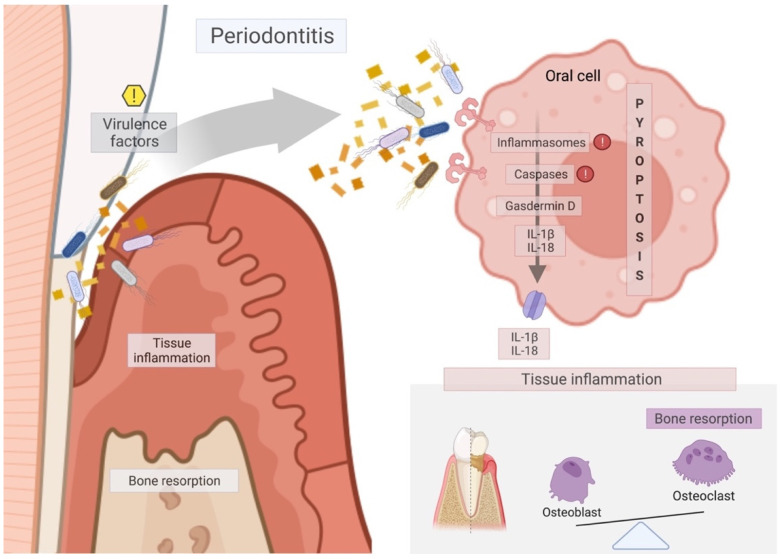
Schematic diagram of the mechanism of pyroptosis pathway on periodontal tissues to the promotion of periodontitis. Virulence factors activate the inflammasome/caspase downstream to the cleavage of Gasdermin D, which is responsible for membrane pore formation and, thus, the release of interleukins IL-1β and IL-18 to the extracellular environment. Those interleukins lead to tissue inflammation and disruption of the balance between bone formation by the osteoblasts and bone resorption by the osteoclasts, thus aggravating the process of periodontitis through soft tissue inflammation (swelling, bleeding) and marginal bone loss. Red exclamation marks mean the main targets for pyroptosis-specific inhibitors, such as MCC950, Ac-YVAD-CHO, Z-LEVD-FMK, and VX765. Yellow exclamation mark indicates the main target for therapeutic approaches that should act on the virulence factors responsible for triggering pyroptosis on periodontal tissues.

Periodontal disease is one of the most prevalent infectious human inflammatory diseases, and it is characterised by the inflammatory reaction and the progressive destruction of the tooth-supporting tissues [30]. It is a response to years of prolonged exposure to a polymicrobial community in the gingiva and periodontal pocket [30], as shown in Figure 3. Periodontitis is associated with Gram-negative anaerobic bacteria, such as *P. gingivalis*, *A. actinomycetemcomitans*, *T. denticola*, *F. nucleatum*, *T. forsythia*, among others found in the dental biofilms [2,3,5,8,14,15,18,67,68]. Gram-negative bacteria are specialised in the production of virulence factors that can trigger periodontal disease. Virulence factors are critical in manipulating and exploiting host immune responses, leading to dysbiosis in the oral cavity and periodontitis progression [54].

#### 4.2.1. Clinical and In Vivo Pieces of Evidence of Pyroptosis on the Periodontal Tissues

The team of Bostanci and Belibasakis reported that inflammasomes in gingival tissues were significantly higher in patients with periodontal disease than healthy patients [55]. Immunohistochemistry confirmed, particularly in the periodontal epithelium layer, that the overall intensity of NLRP3 expression was higher in chronic periodontitis and patients with generalised aggressive periodontitis compared to healthy control subjects [91]. Consistently, NLRP3, caspase-1, caspase-4, and IL-18 was more pronounced in the inflammatory gingiva compared to healthy gingiva [92], similar to what was observed in a rat model exposed to *P. gingivalis* LPS, where caspase-11 was also raised [92]. In addition, the removal of *P. gingivalis* from subgingival biofilms led to the restored expression of NLRP3 and IL-1β [55]. In other models, pyroptosis markers, such as GSDMD [1], NLRP3 [3,14], cleaved caspase-1 [3,14], and IL-1β [1,3,14] were upregulated in diseased periodontal tissues compared to healthy controls. Pyroptosis seems to have an impact on alveolar bone too. Loss-of-function of caspase-1 but not of NLRP3 reduced *A. actinomycetemcomitans*-induced bone resorption in mice [69], implying that caspase-1 is instrumental in modulating inflammation caused by the pathogen [5]. Taken together, there is evidence for pyroptosis signaling in inflamed periodontal tissues, and caspase-1 partially mediates inflammatory osteolysis.

#### 4.2.2. In Vitro Research on Pyroptosis on the Periodontal Disease

Studies collected from the search strategy applied herein (Table 1) point to similarities regarding In vitro analyses, especially regarding the induction of pyroptosis via LPS from both *E. coli* and *P. gingivalis*. *E. coli* LPS led to IL-1β and IL-18 secretion, activated NLRP3 and GSDMD, and cleaved caspase-1 in PDLCs [4], which agrees with previous findings of *E. coli* LPS-stimulated PDLCs, leading to the expression of NLRP3 and caspase-1 and IL-1β secretion [6]. Another study found that *P. gingivalis* LPS activated caspase-1 and caspase-11 in HGFs and PDLCs [1]. Likewise, HGFs stimulated with *P. gingivalis* LPS under hypoxia promoted caspase-1 activation and IL-1β maturation, while *E. coli* LPS also enhanced IL-1β maturation under normoxia [14]. Other In vitro research suggests that hypoxia can be used as an activation signal together with a “startup signal” of LPS to complete the entire pyroptosis pathway [14,74]. Furthermore, macrophages obtained from periodontitis patients were stimulated with *E. coli* LPS and *P. gingivalis* LPS while the expression of caspase-4 and IL-1β was seen for the cells stimulated with *E. coli* LPS [7]. Hence, it seems that *E. coli* LPS has stronger effects on pyroptosis or even potentialise *P. gingivalis* LPS effects in vitro. Alternatively, *P. gingivalis* induced pyroptosis of HGFs by activation of caspase-1 and NLRP6 [47]. Additionally, HGFs infected with *T. denticola* activated caspase-4 and released IL-1β [93], and LPS increased caspase-1 and NLRP3 in mesenchymal cells isolated from the umbilical cord [94]. The In vitro studies presented herein focused on LPS and how it affects mesenchymal cells and macrophages (Table 1). There is, however, a lack of evidence on how other virulence factors than LPS affect pyroptosis and how this affects other cell types, such as epithelial cells.

#### 4.2.3. Virulence Factors Associated with Pyroptosis on the Periodontal Disease

Virulence factors impair the epithelial barrier functions and thus allow the bacterial invasion of the gingiva [95]. Virulence factors also support the dissemination of the bacteria via the bloodstream into peripheral tissues [96] and then assist the bacterium to colonize the new environment [97]. Even though most studies comprise LPS, other virulence factors produced by periodontopathogens are outer membrane vesicles (OMVs), fimbriae, capsules, gingipains, and leukotoxin (LtxA), among many others. They all have roles in regulating immune responses during periodontitis progression [54,98].

OMVs produced by *P. gingivalis* can penetrate host tissues and interact with monocytes and macrophages, inducing strong pro-inflammatory responses, IL-1β secretion, and inflammatory cell death via inflammasome activation [15,16]. Periodontal OMVs produced by *A. actinomycetemcomitans* were internalised into the perinuclear region of HGFs and triggered the innate immunity via carriage of NOD1- and NOD2-active PAMPs [99]. Proteomics of OMVs by *A. actinomycetemcomitans* affirmed the role of such OMVs in periodontal and systemic diseases [100]. *E. coli* OMVs act as a delivery system for cytosolic LPS, which binds and activates cytosolic caspases-11, -4, and -5 to trigger caspase-1-independent pyroptosis through the cleavage of the pore-forming GSDMD [101]. Additionally, OMVs from *T. denticola* and *T. forsythia* can promote disease progression [15].

Fimbriae and capsules can adhere to other bacteria, host tissues, and cells to promote biofilm formation [54]. *E. coli* fimbriae increased IL-1β release from neutrophils involving caspase-1 and NLRP3 activation and stimulated the antimicrobial activity of human neutrophils against *E. coli* [102].

Gingipains provide *P. gingivalis* with the ability to evade host immune responses and clearance, especially through the degradation of extracellular matrix components. *P. gingivalis* strains KDP136 (gingipain-null mutant) or KDP150 (FimA-deficient mutant) are also less pathogenic with respect to NLRP3 activation compared to the original WT strains [96], while NLRP3 activation can also occur in a gingipain-independent manner [103]. Moreover, gingipains enhance the interactions of *P. gingivalis* with other periodontal pathogens [54].

LtxA of *A. actinomycetemcomitans* affects leukocyte populations by activating neutrophil degranulation, causing a massive release of lysosomal enzymes, net-like structures, and matrix metalloproteinases (MMP) and by the induction of apoptosis in lymphocytes [104]. The inhibition of caspase-1 prevents LtxA-mediated cell death in monocytes, suggesting a critical role of pyroptosis to its excecution [105]. *A. actinomycetemcomitans* may also enhance NLRP3 inflammasome expression, irrespective of its major virulence factors [106].

There is a mutual interaction of different virulence factors from different types of bacteria in a coordinated manner [98,104]. Thus, care should be taken when interpreting the observations made of a single virulence factor. Indeed, the complexity of the subgingival biofilm to modulate NLRP3 and IL-1β in cells would require a simulated biofilm in vitro [107]. For instance, based on biofilm research, we can learn that *P. gingivalis* activates the inflammasome to produce IL-1β, whereas others state that *P. gingivalis* inhibits the inflammasome [108]. Future research should thus consider the complexity of the native biofilm with its large spectrum of virulence factors originating from *P. gingivalis*, *A. actinomycetemcomitans*, *T. denticola*, *F. nucleatum*, *T. forsythia*, and other microbial pathogens of the oral biofilm, with respect to the initiation and propagation of pyroptosis.

Responding rapidly to microbial PAMPs and DAMPs is critical to our innate immune system [31,34,68]. Nevertheless, some non-bacterial related issues may act on the activation of pyroptosis [109]. Clinically, aseptic loosening of artificial joint prostheses is the principal reason that limits the long-term use of this type of rehabilitation. Corrosion products activate macrophages to produce pro-inflammatory cytokines, resulting in local osteolysis [5,86,109], while the wear-induced osteolysis is functionally linked to the NALP3 inflammasome [110,111]. Therefore, it is not exclusively the bacterial virulence factors that activate the inflammasome.

Special attention should be taken since periodontitis was found to exacerbate several systemic diseases, including diabetes [112], cardiovascular disease [113], cancer [114], Alzheimer’s disease [115], and other degenerative diseases [116], suggesting a mechanism that involves the dissemination of periodontal pathogens, producing pyroptosis-initiating virulence factors outside the periodontium.

#### 4.2.4. Systemic Disorders Associated with the Periodontal Disease through Pyroptosis

Increasing evidence suggests an association of periodontitis and its keystone pathogen, *P. gingivalis*, with various diseases. For instance, *P. gingivalis* was found to be related with atherosclerosis due to the pyroptosis-related release of IL-1β [17]. In cardiovascular disease, disseminated periopathogens potentially causes the progression of atheroma lesions [83]. For example, caspase-11-gasdermin D-mediated pyroptosis and the subsequent pro-inflammatory response in macrophages are involved in the pathogenesis of atherosclerosis [117], and the selective NLRP3 inhibitor MCC950 hinders atherosclerosis development [118]. When focusing on rheumatoid arthritis, the disseminated *P. gingivalis* and *A. actinomycetemcomitans* may enhance pyroptosis of synovial cells [84]. Early periodontitis may also worsen clinical symptoms in patients with Crohn’s disease [85] as *Porphyromonas* strains were identified in the colonic mucosa of patients with ulcerative colitis and Crohn’s disease [119]. Taken together, there is reason to assume that periopathogens do not exclusively provoke pyroptosis in periodontal tissues. Periopathogens can disseminate into ectopic sites where they potentially exert their deteriorative activity through pyroptosis activation. Thus, targeting pyroptosis in periodontitis is likely to impact systemic health.

Furthermore, the inflammasomes were also found to be the link among endometriosis, atherosclerosis, periodic fever syndromes, vitiligo, Crohn’s disease, gout, asbestosis, silicosis, and periodontitis [70]. In addition, neuroinflammation with pyroptosis is recognised as a pathological factor in Alzheimer’s disease [81,82]. Such associations give clues regarding the pathogenic mechanisms involving inflammasomes that are crucial for developing therapies or even for preventing such diseases [70]. Pyroptosis, thus, becomes a target to prevent systemic inflammatory disorders.

#### 4.2.5. Therapeutic Approaches for Pyroptosis-Related Periodontal Disease

The application of pyroptosis inhibitors has been the focus of recent research. MCC950, an NLRP3 specific inhibitor, restored the expression of osteogenic differentiation markers in cells exposed to *E. coli* LPS [10]. Similarly, VX765, a caspase-1 inhibitor, reduced the expressions of IL-1β, in PDLCs stimulated with *E. coli* LPS or *P. gingivalis* LPS, and decreased the inflammatory responses during periodontitis in vivo [3]. Moreover, Z-LEVD-FMK, a caspase-4 specific inhibitor, led to inhibition of GSDMD cleavage, caspase-4 activation, and IL-1β release in a periodontitis rat model [2]. The inhibition of pyroptosis can be indirect as well. For example, by inhibiting cyclin-dependent kinase 9, flavopiridol dampened pyroptosis in the liver and decreased cell death in LPS-exposed monocytes [120]. Additionally, eldecalcitol, a vitamin D analogue, reduced LPS-induced NLRP3 inflammasome-dependent pyroptosis in HGFs via the Nrf2/HO-1 pathway [121]. Thus, direct and indirect pyroptosis inhibitors could help combating periodontal disease [2]. Pharmacological blocking of pyroptosis, however, should be seen with caution as its inhibition must be balanced against its benefits to strengthen the immune system. Clinically, it seems more realistic to remove pathogens and their virulence factors from the periodontal pockets and thereby reduce, or even prevent, pyroptosis-mediated inflammation and tissue damage. In support of the previous affirmation, professional use of local antimicrobial agents, in conjunction with scaling and root debridement, provides significant benefits in periodontal therapy [122]. Moreover, reducing the microbial charge lowers the chance of disseminating periodontal pathogens and their virulence factors into the periphery [114]. Based on this concept, avoiding the dissemination of oral pathogens supports systemic health, avoiding pyroptosis-mediated inflammation and tissue damage.

## 5. Conclusions

The clinical exacerbated inflammatory processes in periodontitis are yet to be fully understood. The collected data highlights pathways for inflammatory responses that could lead to exacerbated tissue damage and non-responsive therapies in periodontal disease. Pyroptosis is likely to be one of those pathways. Overall, the studies agree that some virulence factors trigger the inflammasome route of caspase-1 activation, which is able to cleave gasdermin D and is also responsible for the maturation and release of interleukins, specifically IL-1β and IL-18. Therefore, pyroptosis is a potential target for periodontal therapy. However, since pyroptosis mainly occurs as a consequence of virulence factors produced by oral pathogens, maintaining oral hygiene is presumably the best strategy to prevent periodontal tissues from pyroptosis-mediated tissue destruction. Finally, it is also important to keep in mind the potential beneficial effects of reducing other inflammatory diseases that are linked with the dissemination of oral pathogens.

## Figures and Tables

**Table 1:** Included experimental articles.

	Authors	Year	Journal	Study Type	Methods	Outcomes	Title and Reference
1	Zhang X, He S, Lu W, Lin L, Xiao H.	2021	In Vitro Cellular & Developmental Biololy-Animal	In Vitro	PDLCs were stimulated with *E. coli* LPS (1, 5, and 10 µg/mL for 6 h and 12 h). GCF were collected from periodontitis patients and healthy volunteers.	LPS suppressed PDLCs viability and led to production and secretion of IL-1β, IL-18, IL-6, and TNF-α in a time- and concentration-dependent manner. LPS activated NLRP3 and GSDMD, cleaved caspase-1, and upregulated GSK-3β. Blockage of GSK-3β restrained NLRP3-mediated pyroptosis. Pro-inflammatory cytokines were upregulated in periodontal patients’ GCF but not in healthy volunteers.	Glycogen synthase kinase-3β (GSK-3β) deficiency inactivates the NLRP3 inflammasome-mediated cell pyroptosis in LPS-treated periodontal ligament cells (PDLCs) [4]
2	Oka S, Li X, Sato F, Zhang F, Tewari N, Kim I-S, Zhong L, Hamada N, et al.	2021	Journal of Periodontal Research	In vitro and in vivo	HGFs and PDLCs were stimulated with *P. gingivalis* LPS (10 µg/mL for 24 h). Mouse experimental periodontitis model (WT and differentiated embryo chondrocyte 2 (Dec2) KO) was established.	LPS activated caspase-1, caspase-11, and NF-κB. Dec2 KO upregulated LPS-induced pyroptosis, resulting in IL-1β release. The inhibition of Dec2 led to the activation of caspase-1 and GSDMD, reduced the phosphorylation and translocation of NF-κB, decreased IL-1β expression, reducing pyroptosis.	A deficiency of Dec2 triggers periodontal inflammation and pyroptosis [1]
3	Chen Q, Cao M, Ge H.	2021	BioMed Research International	In Vitro	PDLCs were treated with *P. gingivalis* LPS (100 ng/mL for 72 h). The expression of metastasis-associated lung adenocarcinoma transcript 1 (MALAT1) and miR-769-5p in gingival tissues of patients with periodontitis and LPS-treated PDLCs was evaluated.	MALAT1 KO promoted cell viability and inhibited inflammation and pyroptosis. The expression of MALAT1 and hypoxia-inducible factor 3A (HIF3A) was enhanced, and the expression of miR-769-5p was reduced in gingival tissues of patients with periodontitis and LPS-treated PDLCs.	Knockdown of MALAT1 inhibits the progression of chronic periodontitis via targeting miR-769-5p/HIF3A axis [13]
4	Liu S, Du J, Li D, Yang P, Kou Y, Li C, Zhou Q, Lu Y, et al.	2020	Journal of Molecular Histology	In Vitro	Human osteoblast-like cells were exposed to *E. coli* LPS (0.5, 1, or 2 μg/mL) for 24 h and 48 h. *N*-acetyl-*L*-cysteine (NAC) was used to decrease the intracellular ROS level and MCC950 was used to inhibit pyroptosis.	LPS led to NLRP3-mediated pyroptosis in a time- and dose-dependent manner. The inhibition of ROS with NAC attenuated oxidative stress-mediated pyroptosis. The inhibition of pyroptosis with MCC950 restored the expression of osteogenic differentiation-related proteins of osteoblasts.	Oxidative stress induced pyroptosis leads to osteogenic dysfunction of MG63 cells [10]
5	Cheng R, Feng Y, Zhang R, Liu W, Lei L, Hu T.	2018	Biochimica et Biophysica Acta—Molecular Basis of Disease	In vitro and in vivo	PDLCs were stimulated with *E. coli* LPS (1 μg/mL) or *P. gingivalis* LPS (10 μg/mL) for 24 h. Rat experimental periodontitis model was established. VX765 caspase-1 inhibitor was used to block pyroptosis.	VX765 inhibited the expressions of IL-1β, monocyte chemoattractant protein-1 (MCP-1), IL-6, and IL-8 In vitro, decreasing inflammatory responses during periodontitis. VX765 suppressed bone loss in vivo, linking pyroptosis to bone resorption in acute apical periodontitis.	The extent of pyroptosis varies in different stages of apical periodontitis [3]
6	Chen R, Liu W, Zhang R, Feng Y, Bhowmick NA, Hu T.	2017	Frontiers in Cellular and Infection Microbiology	In vitro and in vivo	HGFs were stimulated with *E. coli* LPS (1 μg/mL) or *P. gingivalis* LPS (10 μg/mL) at 2% or 20% O_2_ for 6 h. Mouse experimental periodontitis model was established.	*P. gingivalis* LPS slightly decreased the level of NLRP3 and IL-1β under normoxia. Hypoxia reversed the effects of *P. gingivalis* LPS, promoting caspase-1 activation and IL-1β maturation. *E. coli* LPS enhanced IL-1β maturation in both normoxia and hypoxia, and turned normoxia into hypoxia in the periodontitis model, suggesting to increase the inflammatory effect of *P. gingivalis* LPS.	*Porphyromonas gingivalis*-derived lipopolysaccharide combines hypoxia to induce caspase-1 activation in periodontitis [14]
7	Cecil JD, O’Brien-Simpson NM, Lenzo JC, Holden JA, Singleton W, Perez-Gonzalez A, Mansell A, Reynolds EC.	2017	Frontiers in Immunology	In vitro and in vivo	THP-1 (monocytes) and macrophages extracted from C57BL/6 J mice (ex vivo and in vivo) were treated with intraperitoneal injections of *P. gingivalis*, *T. denticola*, and *T. forsythia* OMVs (100 ng protein/mL) for 4 h. Cells were stimulated with nigericin (10 µM), silica (125 mg/mL), or transfected with poly(dAdT) (250 ng/mL) using lipofectamine LTX for 6 h.	OMVs interacted with monocytes and macrophages, inducing phagocytosis, NF-κB activation, IL-1β secretion, and cell death via NLRP3 activation. The immune stimulatory effects of *P. gingivalis* OMVs are suggested to dysregulate the host immune response and initiate the disease, while the pro-inflammatory effects of *T. denticola* and *T. forsythia* OMVs are suggested to promote disease progression.	Outer membrane vesicles prime and activate macrophage inflammasomes and cytokine secretion In vitro and in vivo [15]
8	Fleetwood AJ, Lee MKS, Singleton W, Achutan A, Lee M-C, O’Brien-Simpson NM, Cook AD, Murphy AJ, et al.	2017	Frontiers in Cellular and Infection Microbiology	In vitro and in vivo	C57BL/6 mouse and human macrophages were treated with viable *P. gingivalis*, heat-killed *P. gingivalis*, OMVs, or heat-inactivated OMVs at a MOI of 10:1, 25:1 (protein concentration of about 3.0 μg/mL) or 100:1 bacilli or OMVs/cell for 2 h.	*P. gingivalis* did not lead to the activation of NLRP3 while *P. gingivalis* OMVs activated caspase-1, produced large amounts of IL-1β and IL-18, released lactate dehydrogenase (LDH), and were positive for 7-amino actinomycin D (7-AAD) staining, thus indicating of pyroptosis.	Metabolic remodeling, inflammasome activation, and pyroptosis in macrophages stimulated by *Porphyromonas gingivalis* and its outer membrane vesicles [16]
9	Lu WL, Song DZ, Yue JL, Wang TT, Zhou XD, Zhang P, Zhang L, Huang DM.	2017	International Endodontic Journal	In Vitro	PDLCs were stimulated with MDP (10 μg/mL) for 0, 1, 3, 8, 14 or 24 h; *E. coli* LPS (0.5 μg/mL) for 0, 4, 8 or 24 h; or MDP and LPS in combination for 0, 4, 8 or 24 h.	MDP, LPS, or MDP in combination with LPS promoted the expression of NLRP3, caspase-1, and induced IL-1β secretion. MDP exhibited synergistic or additive effects with LPS to upregulate the expression of NLRP3, ASC and caspase-1.	NLRP3 inflammasome may regulate inflammatory response of human periodontal ligament fibroblasts in an apoptosis-associated speck-like protein containing a CARD (ASC)-dependent manner [6]
10	Brown PM, Kennedy DJ, Morton RE, Febbraio M.	2015	PLoS ONE	In Vivo	Cd36/Ldlr and Ldlr mice were derived from a cross between Cd36° and Ldlr mice. *P. gingivalis* (~2 × 10^9^ CFU/mL) were resuspended in saline containing 2% carboxymethylcellulose (as a thickener to promote adherence) prior to oral inoculation of mice.	An increase of 225% (females) and 175% (males) was found in periodontal lesions compared to uninfected mice. This increase was CD36/SR-B2-dependent since there was no significant change in lesion burden between infected and uninfected Cd36°/Ldlr mice. Activation of the NLRP3 by *P. gingivalis* is mediated by CD36/SR-B2 and TLR2, leading to systemic release of IL-1β and inducing pyroptosis.	CD36/SR-B2-TLR2 dependent pathways enhance *Porphyromonas gingivalis* mediated atherosclerosis in the Ldlr KO mouse model [17]
11	Taxman DJ, Swanson KV, Broglie PM, Wen H, Holley-Guthrie E, Huang MT-H, Callaway JB, Eitas TK, et al.	2012	Journal of Biological Chemistry	In vitro and in vivo	*MyD88*^−/−^, *Nlrp3*^−/−^, *Asc*^−/−^, and *Casp1*^−/−^ mice macrophages were infected with *P. gingivalis*. Macrophages were stimulated with *E. coli* LPS (1 μg/mL) for 3 h, followed by ATP (2 mM) for 0.5 h, nigericin (20 μM) for 0.5 h, monosodium urate (200 μg/mL) for 6 h, alum crystals (400 μg/mL) for 6 h, or *S. aureus* peptidoglycan (20 μg/mL) for 14-16 h.	*P. gingivalis* lacks signaling capability for the NLRP3 activation and can suppress NLRP3 activation by *F. nucleatum*, thus repressing IL-1β and IL-18 release and cell death. *P. gingivalis* can repress NLRP3 activation by *E. coli*, and by DAMPs and PAMPs that mediate activation through endocytosis, but cannot suppress NLRP3 activation by ATP or nigericin, suggesting that *P. gingivalis* preferentially suppress endocytic pathways towards NLRP3 activation.	*Porphyromonas gingivalis* mediates inflammasome repression in polymicrobial cultures through a novel mechanism involving reduced endocytosis [18]
12	Domon H, Takahashi N, Honda T, Nakajima T, Tabeta K, Abiko Y, Yamazaki K.	2009	Clinica Chimica Acta	In Vitro	Cells were obtained from human periodontitis patients. Macrophages were stimulated with *E. coli* LPS (1 µg/mL), *P. gingivalis* LPS (1 µg/mL), IFN-γ (100 or 500 U), or tunicamycin (1 μg/mL) for 1, 3, 6, 12, or 24 h. The expression of unfolded protein response (UPR) was analysed.	The expression of UPR-related genes was higher in periodontitis than in gingivitis lesions. *P. gingivalis* LPS (but not *E. coli* LPS or IFN-γ) failed to up-regulate gene expressions. Macrophages stimulated with *E. coli* LPS or IFN-γ expressed IL-β and caspase-4 at the gene level while tunicamycin did not.	Up-regulation of the endoplasmic reticulum stress-response in periodontal disease [7]

**Abbreviations:** Colony forming units (CFU); Gasdermin D (GSDMD); Gingival crevicular fluid (GCF); Glycogen synthase kinase-3β (GSK-3β); Human gingival fibroblasts (HGFs); Interleukin (IL); Knockout (KO); Lipopolysaccharides (LPS); Multiplicity of infection (MOI); Nuclear factor kappa B (NF-kB); Outer membrane vesicles (OMVs); Pathogen-associated molecular patterns (PAMPs); Periodontal ligament (PDL); Primary human periodontal ligament cells (PDLCs); Reactive oxygen species (ROS); Wild-type (WT).

**Table 2:** Literature review article.

Authors	Year	Journal	Main Findings	Title and Reference
De Andrade KQ, Almeida-da-Silva CLC, Coutinho-Silva R.	2017	Mediators of Inflammation	Inflammasomes are involved in the pathogenesis of periodontitis; however, it is necessary to determine which inflammasomes, others than the typical NLRP3, contribute to the pathogenesis of periodontitis induced by *P. gingivalis* and *F. nucleatum*. With more solid literature on the signaling pathways and immune responses during infection with these bacteria, more effective treatments for periodontitis may appear.	Immunological pathways triggered by *Porphyromonas gingivalis* and *Fusobacterium nucleatum*: therapeutic possibilities? [8]

**Table 3:** Excluded articles.

	Authors	Year	Journal	Type of Study	Reason of Exclusion	Title and Reference
1	Wang J, Du, C, Xu L.	2021	Archives of Oral Biology	In Vitro	Studied apoptosis	Circ_0081572 inhibits the progression of periodontitis through regulating the miR-378h/RorA axis [19]
2	Liu P, Cui, L, Shen L.	2020	Bioscience Reports	In Vitro	Studied apoptosis	Knockdown of TRIM52 alleviates LPS-induced inflammatory injury in human periodontal ligament cells through the TLR4/NF-κB pathway [20]
3	Zhang K, He S, Dai Z, Cao L, Yue S, Bai Y, Zheng M.	2020	Archives of Oral Biology	In Vitro	Studied apoptosis	Axin 1 knockdown inhibits osteoblastic apoptosis induced by Porphyromonas gingivalis lipopolysaccharide [21]
4	Zhou Y, Zhang H, Zhang G, He Y, Zhang P, Sun Z, Gao Y, Tan Y.	2018	Molecular Medicine Reports	In Vitro	Studied apoptosis	Calcitonin gene-related peptide reduces Porphyromonas gingivalis LPS-induced TNF-α release and apoptosis in osteoblasts [22]
5	Shirasugi M, Nishioka K, Yamamoto T, Nakaya T, Kanamura N.	2017	Biochemical and Biophysical Research Communications	In Vitro	Studied apoptosis and cytostasis	Normal human gingival fibroblasts undergo cytostasis and apoptosis after long-term exposure to butyric acid [23]
6	Zhu X, Lu W, Chen Y, Cheng X, Qiu J, Xu Y, Sun Y.	2016	PLoS ONE	In Vitro	Studied apoptosis	Effects of *Porphyromonas gingivalis* Lipopolysaccharide olerized monocytes on inflammatory responses in neutrophils [24]
7	Deepak V, Kasonga A, Kruger MC, Coetzee M.	2016	Biological and Pharmaceutical Bulletin	In Vitro	Studied apoptosis	Carvacrol inhibits osteoclastogenesis and negatively regulates the survival of mature osteoclasts [25]
8	Jönsson D, Nilsson B-O.	2012	Journal of Periodontal Research	In Vitro	Studied apoptosis	The antimicrobial peptide LL-37 is anti-inflammatory and proapoptotic in human periodontal ligament cells [26]
9	Zaric, S, Shelburne C, Darveau R, Quinn DJ, Weldon S, Taggart CC, Coulter WA.	2010	Infection and Immunity	In Vitro	Studied apoptosis	Impaired immune tolerance to *Porphyromonas gingivalis* lipopolysaccharide promotes neutrophil migration and decreased apoptosis [27]
10	Thammasitboon K, Goldring SR, Boch JA.	2006	Bone	In Vitro	Studied apoptosis	Role of macrophages in LPS-induced osteoblast and PDL cell apoptosis [28]

## Data Availability

The data that support the findings of this study are available from the corresponding author upon reasonable request.
